# Supplementary material for: How many days of continuous physical activity monitoring reliably represent time in different intensities in cancer survivors
Source: PLoS One. 2023 Apr 24;18(4):e0284881. doi: 10.1371/journal.pone.0284881 (PMC10124860; doi:10.1371/journal.pone.0284881)
Supplement: S1 File — (DOCX) [file pone.0284881.s001.docx]

**Supplementary material**

Table 1**.** Comparisons of the three ICC models for each intensity in the sample of 736 cancer survivors.

|  |  |  |  |
| --- | --- | --- | --- |
|  | One-way random  ICC(1) | Two-way random absolute agreement  ICC(A,1) | Two-way random consistency  ICC(C,1) |
| LPA | 0.690 | 0.690 | 0.690 |
| MPA | 0.606 | 0.606 | 0.606 |
| VPA | 0.378 | 0.378 | 0.379 |
| MVPA | 0.610 | 0.610 | 0.610 |
| ICC, Intra-class Correlation Coefficient; LPA, Light intensity Physical Activity; MPA, Moderate intensity Physical Activity; VPA, Vigorous intensity Physical Activity; MVPA, Moderate-to-Vigorous intensity Physical Activity. | | | |

Table 2. Mean daily time spent in different physical activity intensities for the sample with six valid monitoring days, and stratified on participant characteristics. Independent sample t-tests and one-way ANOVA with Bonferroni post-hoc tests were used to assess differences in physical activity.

|  | |  | | |  | | |  | | |  | | |
| --- | --- | --- | --- | --- | --- | --- | --- | --- | --- | --- | --- | --- | --- |
| Mean daily physical activity | | Light intensity | | | Moderate intensity | | | Vigorous intensity | | | MVPA | | |
|  | n | Mean minutes (SD) | CV% | Relative SEM% | Mean minutes (SD) | CV% | Relative SEM% | Mean minutes (SD) | CV% | Relative SEM% | Mean minutes(SD) | CV% | Relative SEM% |
| All | 736 | 220.49 (81.97) | 37.18 | 1.37 | 75.99 (54.19) | 71.31 | 2.63 | 3.43 (7.48) | 218.04 | 8.04 | 79.42 (57.29) | 72.14 | 2.66 |
| Male | 259 | 185.50 (63.51)** | 34.23 | 2.13 | 88.14 (59.44)** | 67.45 | 4.19 | 3.31 (6.35) | 191.61 | 11.91 | 91.45 (62.18)** | 67.99 | 4.22 |
| Female | 464 | 239.51 (84.79) | 35.40 | 1.64 | 69.11 (49.80) | 72.07 | 3.35 | 3.42 (7.99) | 233.87 | 10.86 | 72.52 (53.19) | 73.34 | 3.40 |
| Breast cancer | 289 | 256.79 (86.67)**^a,b^ | 33.75 | 1.99 | 64.90 (43.64) | 67.25 | 3.96 | 3.99 (8.48) | 212.62 | 12.51 | 68.89 (47.44) | 68.87 | 4.05 |
| Colorectal cancer | 366 | 196.82 (67.32) | 34.21 | 1.79 | 82.97 (58.75)**^a^ | 70.81 | 3.70 | 2.79 (6.50) | 233.03 | 12.18 | 85.76 (61.74)*^a^ | 71.99 | 3.76 |
| Prostate cancer | 72 | 192.09 (77.1) | 40.14 | 4.73 | 82.86 (60.84)*^b^ | 73.43 | 8.65 | 3.78 (7.14) | 188.97 | 22.27 | 86.63 (63.38) | 73.16 | 8.62 |
| <60 years | 250 | 255.34 (90.72)** | 35.53 | 2.25 | 82.06 (52.83)* | 64.38 | 4.07 | 5.00 (9.19)** | 183.74 | 11.62 | 87.06 (56.63)* | 65.05 | 4.11 |
| ≥ 60 years | 473 | 201.57 (70.28) | 34.87 | 1.60 | 72.68 (54.67) | 75.23 | 3.46 | 2.52 (6.16) | 244.33 | 11.23 | 75.20 (57.22) | 76.09 | 3.50 |
| Low-medium education | 301 | 214.39 (75.72) | 35.32 | 2.04 | 73.48 (54.72) | 74.47 | 4.29 | 2.69 (7.23) | 269.30 | 15.52 | 76.16 (57.9) | 76.02 | 4.38 |
| Higher education | 402 | 223.12 (85.39) | 38.27 | 1.91 | 77.89 (53.03) | 68.01 | 3.40 | 3.83 (7.45)* | 194.46 | 9.70 | 81.72 (55.97) | 68.48 | 3.42 |
| BMI <25 | 292 | 250.50 (82.01)**^c,d^ | 32.74 | 1.92 | 93.62 (58.46)**^c,d^ | 62.44 | 3.65 | 5.31 (9.03)**^c,d^ | 169.88 | 9.94 | 98.93 (61.43)**^c,d^ | 62.09 | 3.63 |
| BMI ≥25 <30 | 280 | 206.74 (72.78)**^e^ | 35.21 | 2.10 | 72.55 (48.98)**^e^ | 67.51 | 4.03 | 2.19 (5.19) | 237.55 | 14.20 | 74.74 (51.64)**^e^ | 69.09 | 4.13 |
| BMI ≥30 | 130 | 172.14 (68.59) | 39.85 | 3.49 | 44.61 (36.60) | 82.04 | 7.20 | 1.11 (5.62) | 504.60 | 44.26 | 45.73 (37.73) | 82.52 | 7.24 |
| * P<0.05 **p<0.001.  BMI, Body Mass Index; SD, Standard Deviation; SEM, Standard Error of the Mean; CV, Coefficient of Variation; MVPA, Moderate-to-Vigorous intensity Physical Activity.  ^a^=breast cancer compared to colon cancer; ^b^=breast cancer compared to prostate cancer; ^c^=BMI<25 compared to BMI ≥25 <30; ^d^= BMI<25 compared to BMI ≥30; ^e^= BMI ≥25 <30 compared to BMI ≥30. | | | | | | | | | | | | | |

Table 3. Variance estimates from the ICC(A,1) for minutes spent in each physical activity intensity. Bias constituted <1% of the total variation for all groups. Inter-individual variance contribution expressed as percent represents the ICC(A,1) for one monitoring day.

|  |  |  |  |  |  |
| --- | --- | --- | --- | --- | --- |
| **Light intensity physical activity** | ***n*** | **Total variance** | **Inter-individual variance contribution (%)** | **Intra-individual variance contribution (%)** | **Bias contribution to total variance (%)** |
| All | 736 | 9060.73 | 69.00 | 30.94 | 0.06 |
| Male | 259 | 5795.00 | 63.54 | 36.31 | 0.15 |
| Female | 464 | 9851.15 | 67.58 | 32.35 | 0.07 |
| Breast cancer | 289 | 10269.53 | 67.76 | 32.25 | -0.01 |
| Colorectal cancer | 366 | 6646.14 | 61.87 | 38.01 | 0.12 |
| Prostate cancer | 72 | 7697.33 | 72.79 | 26.66 | 0.56 |
| <60 years | 250 | 11039.65 | 69.48 | 30.46 | 0.06 |
| ≥ 60 years | 473 | 7030.97 | 64.32 | 35.61 | 0.07 |
| Low-medium education | 301 | 8272.41 | 63.20 | 36.62 | 0.18 |
| Higher education | 402 | 9479.29 | 72.31 | 27.68 | 0.01 |
| BMI <25 | 292 | 9165.84 | 68.07 | 31.84 | 0.09 |
| BMI ≥25 <30 | 280 | 7539.36 | 64.33 | 35.57 | 0.09 |
| BMI ≥30 | 130 | 6839.60 | 62.59 | 37.24 | 0.18 |
| **Moderate intensity physical activity** |  |  |  |  |  |
| All | 736 | 4371.67 | 60.61 | 39.37 | 0.03 |
| Male | 259 | 5441.93 | 57.92 | 42.06 | 0.02 |
| Female | 464 | 3651.91 | 61.50 | 38.53 | -0.03 |
| Breast cancer | 289 | 3025.65 | 55.52 | 44.60 | -0.12 |
| Colorectal cancer | 366 | 5053.20 | 61.99 | 37.91 | 0.10 |
| Prostate cancer | 72 | 5532.45 | 60.30 | 39.61 | 0.09 |
| <60 years | 250 | 4310.73 | 57.71 | 42.26 | 0.03 |
| ≥ 60 years | 473 | 4380.47 | 61.89 | 38.10 | 0.01 |
| Low-medium education | 301 | 4471.89 | 60.35 | 39.63 | 0.02 |
| Higher education | 402 | 4209.07 | 60.21 | 39.67 | 0.11 |
| BMI <25 | 292 | 5140.47 | 59.76 | 40.31 | -0.07 |
| BMI ≥25 <30 | 280 | 3812.43 | 55.56 | 44.23 | 0.21 |
| BMI ≥30 | 130 | 2195.24 | 53.22 | 46.84 | -0.07 |
| **Vigorous intensity physical activity** |  |  |  |  |  |
| All | 736 | 116.35 | 37.80 | 62.06 | 0.15 |
| Male | 259 | 100.02 | 28.44 | 71.32 | 0.23 |
| Female | 464 | 120.84 | 43.39 | 56.57 | 0.04 |
| Breast cancer | 289 | 139.06 | 42.09 | 57.73 | 0.17 |
| Colorectal cancer | 366 | 91.13 | 35.61 | 64.51 | -0.12 |
| Prostate cancer | 72 | 115.26 | 33.13 | 66.55 | 0.33 |
| <60 years | 250 | 166.45 | 40.92 | 58.79 | 0.29 |
| ≥ 60 years | 473 | 83.26 | 34.76 | 65.26 | -0.02 |
| Low-medium education | 301 | 105.12 | 39.70 | 60.34 | -0.04 |
| Higher education | 402 | 116.43 | 37.28 | 62.42 | 0.30 |
| BMI <25 | 292 | 172.68 | 36.69 | 62.92 | 0.39 |
| BMI ≥25 <30 | 280 | 66.30 | 28.79 | 71.09 | 0.12 |
| BMI ≥30 | 130 | 52.12 | 52.57 | 47.54 | -0.11 |
| **MVPA** |  |  |  |  |  |
| All | 736 | 4866.57 | 60.95 | 39.00 | 0.05 |
| Male | 259 | 5985.10 | 57.53 | 42.40 | 0.06 |
| Female | 464 | 4114.50 | 62.51 | 37.53 | -0.03 |
| Breast cancer | 289 | 3475.41 | 57.70 | 42.40 | -0.10 |
| Colorectal cancer | 366 | 5606.26 | 61.60 | 38.32 | 0.08 |
| Prostate cancer | 72 | 5941.32 | 61.16 | 38.71 | 0.13 |
| <60 years | 250 | 4881.61 | 58.86 | 41.04 | 0.10 |
| ≥ 60 years | 473 | 4810.55 | 61.67 | 38.31 | 0.02 |
| Low-medium education | 301 | 4984.30 | 60.71 | 39.25 | 0.04 |
| Higher education | 402 | 4676.20 | 60.40 | 39.47 | 0.13 |
| BMI <25 | 292 | 5690.17 | 59.56 | 40.49 | -0.05 |
| BMI ≥25 <30 | 280 | 4212.14 | 56.01 | 43.75 | 0.24 |
| BMI ≥30 | 130 | 2355.33 | 52.52 | 47.57 | -0.09 |
| BMI, Body Mass Index; MVPA, Moderate-to-Vigorous intensity Physical Activity; ICC(A,1), Two-way random Intra-class Correlation Coefficient absolute agreement. | | | | | |
