# Supplementary material for: How many days of continuous physical activity monitoring reliably represent time in different intensities in cancer survivors
Source: PLoS One. 2023 Apr 24;18(4):e0284881. doi: 10.1371/journal.pone.0284881 (PMC10124860; doi:10.1371/journal.pone.0284881)
Supplement: Attachment — Submitted filename: Response to Reviewers.docx [file pone.0284881.s003.docx]

# Response to Reviewers

Journal requirements:

1. **Response:** Manuscript has been revised to meet the PLOS ONE style requirements

2. **Response:** An ethic statement has been added to the Methods section of the manuscript.

Review Comments to the Author

Reviewer #1

1. **Comment:** *Why wasn't cancer stage, treatment history, and time since completing treatment considered in the analysis? If the data is available, this analysis and findings should be included. If the data is not available and explanation for why it is not available should be included in the manuscript.*

**Response:** Thank you for the comment. Information about cancer stage, type, and treatment history has been added to the manuscript.

Unfortunately, we were not able to harmonize data on cancer stage, treatment history and time since diagnosis. Detailed information about cancer stage was only available for one of the included studies. In the Phys-Can study, physical activity was monitored before participants started neoadjuvant or adjuvant treatment, however detailed information about where participants were in their treatment trajectory was not available. In the CRC-NORDIET study, all participants had completed surgery, but some received adjuvant chemotherapy with no additional information on the duration/cycles. Thus, some of these participants may have still been receiving chemotherapy when monitored, while others had finished treatment. This heterogeneity complicated stratification on treatment type and time since treatment. However, some additional information on treatment type has been added to the Method section and Strengths and limitations, page 6 lines 106-107 and page 14 lines 278-285.

2. **Comment:** *Conclusions should be revised in order to be specific to the time relevant in the cancer care continuum and cancer type to which this data applies. For example, "Only two days of wear time is needed for LPA in breast, CRC, and prostate cancer survivors at least 2-months post-treatment."*

**Response:** Conclusion has been revised to be specific of the time relevant in the cancer care continuum and cancer type.

Reviewer #2

**Comment:** *There is misunderstanding in the Spearman-Brown formula. K is the ratio of number of new additional days, to the original number of days. So k=6 is actually 6x6=36* *days in total. If the authors want to test how many days within 6 days, k should be 1/6, 2/6, …, 6/6 instead.

The analysis needs to be performed again and conclusions needs to be re-written.*

**Response:**

Thank you for the comment, this is certainly something we should specify clearer. If I understand correctly, I believe you are referring to the use of the Spearman-Brown prophecy formula when assessing the *number of items* in an instrument, and their internal consistency (see de Vet, Mokkink, Mosmuller, and Terwee (2017), section 4).

The Spearman-Brown formula applied in our study assesses *interrater reliability* when the number of raters change, see section 4 and appendix A.4 by de Vet et al. (de Vet et al., 2017). In our case a monitoring day would be considered a “rater”. The formula calculates how many scores (days of measurement) need to be average to achieve acceptable reliability. We used the formula for varying days/raters and obtained new coefficients. It is also possible to rewrite the equation and vary desired ICC and obtain values for number of days instead (Mattocks et al., 2008).

k = [ICC_desired_/(1-ICC_desired_)][(1-ICC_single measure_)/ICC_single measure_]

However, as we wanted to obtain ICCs for whole days, we applied the current equation.

[k*ICC_single measure_]/[1+(k-1)*ICC_single measure_]

In the formula, the coefficient obtained through the ICC analysis for “single measures” (for example 0.690 for LPA) is plotted into the equation and the number of days (k) varied. As our aim was to assess the absolute agreement between physical activity measures from each day, we used the coefficients representing ICC_agreement_.

We have conferred with a statistician (Dr. A. Stenling) and validated our use of the formula against previous research (da Silva et al., 2019; Dillon et al., 2016; Ricardo et al., 2020). If it is of interest, we can send our calculations of the ICC and Spearman-Brown formula.

**Comment**: *To fully understand the variation contributions (intra- or inter), a linear mixed model for longitudinal analysis should be considered. A mean plot by each day and characteristic groups can be illustrated. Furthermore, Bland-Altman plot may be considered too. The authors may consult with a statistician.*

**Response:** We recognize that the ANOVA framework for the ICC has its limitations and that mixed-effect models may be more appropriate in certain settings and conditions, e.g. negative or zero ICC, missing repeated data, confounding effects, sampling error, or large datasets difficult to assess in a limited spreadsheet environment (Chen et al., 2018; Pleil, Wallace, Stiegel, & Funk, 2018). Also, with complex assessments with numerous factors influencing the variance of the assessments, mixed models may be especially relevant. We consider our data to be less subject to such impacts as it is a rather simple analysis of the variance in measured physical activity level with no missing data.

Previous research comparing spreadsheet calculations, ANOVA and a mixed model approach with both balanced data containing the same number of repeated measures and no missing values, as well as unbalanced data with fewer subjects, resulted in essentially the same ICC estimates. The method of calculation was found to be irrelevant for the simulated data (Pleil et al., 2018).

While Bland-Altman plots can support visual interpretations of variance between two measures, primarily when comparing with a gold standard method, we do not believe it would contribute to further understanding of our variance estimates. If we were to compare each combination of days against the mean of six days, (as has been previously published (da Silva et al., 2019)) it would require 20 Bland-Altman plots (5 plots for combinations of days times 4 different intensities). Furthermore, while six days served as the criterion, we do not have a defined “gold standard” for number of measuring days, as coefficients for higher number of days may have only small variations in ICC, and not differ from the criterion.

A modified Bland-Altman plot is possible to create for obtaining one plot within each intensity (4 plots in total), however, the interpretation of these differs slightly from the traditional Bland-Altman plots (Möller, Debrabant, Halekoh, Petersen, & Gerke, 2021). Thus, we regard our choice of method as sufficient for the purpose of the current study.

**Comment:** *ICC better presented with 95% confidence intervals.*

**Response:** Confidence intervals have been added to the ICC results.

**Comment:** *In several places, “Analyzes” should be “Analyses”.*

**Response:** This has been changed.

**References:**

Chen, G., Taylor, P. A., Haller, S. P., Kircanski, K., Stoddard, J., Pine, D. S., . . . Cox, R. W. (2018). Intraclass correlation: Improved modeling approaches and applications for neuroimaging. *Human Brain Mapping, 39*(3), 1187-1206. doi:<https://doi.org/10.1002/hbm.23909>

da Silva, S. G., Evenson, K. R., Ekelund, U., da Silva, I. C. M., Domingues, M. R., da Silva, B. G. C., . . . Hallal, P. C. (2019). How many days are needed to estimate wrist-worn accelerometry-assessed physical activity during the second trimester in pregnancy? *PLoS One, 14*(6), e0211442. doi:10.1371/journal.pone.0211442

de Vet, H. C. W., Mokkink, L. B., Mosmuller, D. G., & Terwee, C. B. (2017). Spearman–Brown prophecy formula and Cronbach's alpha: different faces of reliability and opportunities for new applications. *Journal of Clinical Epidemiology, 85*, 45-49. doi:<https://doi.org/10.1016/j.jclinepi.2017.01.013>

Dillon, C. B., Fitzgerald, A. P., Kearney, P. M., Perry, I. J., Rennie, K. L., Kozarski, R., & Phillips, C. M. (2016). Number of Days Required to Estimate Habitual Activity Using Wrist-Worn GENEActiv Accelerometer: A Cross-Sectional Study. *PLoS One, 11*(5), e0109913-e0109913. doi:10.1371/journal.pone.0109913

Mattocks, C., Ness, A., Leary, S., Tilling, K., Blair, S. N., Shield, J., . . . Riddoch, C. (2008). Use of Accelerometers in a Large Field-Based Study of Children: Protocols, Design Issues, and Effects on Precision. *Journal of Physical Activity and Health, 5*(s1), S98-S111. doi:10.1123/jpah.5.s1.s98

Möller, S., Debrabant, B., Halekoh, U., Petersen, A. K., & Gerke, O. (2021). An Extension of the Bland-Altman Plot for Analyzing the Agreement of More than Two Raters. *Diagnostics (Basel), 11*(1). doi:10.3390/diagnostics11010054

Pleil, J. D., Wallace, M. A. G., Stiegel, M. A., & Funk, W. E. (2018). Human biomarker interpretation: the importance of intra-class correlation coefficients (ICC) and their calculations based on mixed models, ANOVA, and variance estimates. *Journal of Toxicology and Environmental Health, Part B, 21*(3), 161-180. doi:10.1080/10937404.2018.1490128

Ricardo, L. I. C., Wendt, A., Galliano, L. M., de Andrade Muller, W., Niño Cruz, G. I., Wehrmeister, F., . . . Crochemore M. Silva, I. (2020). Number of days required to estimate physical activity constructs objectively measured in different age groups: Findings from three Brazilian (Pelotas) population-based birth cohorts. *PLoS One, 15*(1), e0216017. doi:10.1371/journal.pone.0216017
